# On the Treatment of Cough

**Published:** 1891-02-07

**Authors:** 


### THE PRACTITIONER'S MIRROR AND RETROSPECT.

[*The Editor will be glad to receive offers of co-operation and contributions from members of the profession. There is no doubt that much valuable material full of interest and instruction is at present lost to science. This is largely so in the case of* (1) *the younger and less-known members of the hospital staff* (2) *those connected with cottage hospitals, and* (3) *the great body of medical practitioners not attached to any institution. The co-operation of all is cordially invited to enable the Editor to make* THE HOSPITAL *of the utmost possible use and value to the profession. Specialists willing to review books on their special subjects are invited to communicate this fact at once. All letters should be addressed to* THE EDITOR, THE LODGE, PORCHESTER SQUARE, LONDON, W.]

### THE POST-GRADUATE COURSE AT THE BROMPTON CONSUMPTION HOSPITAL.

### ON THE TREATMENT OF COUGH.

Dr. Mitchell Bruce, in commencing his lecture, said the subject of cough was too vast a one for a single lecture, he could only speak of one variety of cough, namely, the excessive cough, which occurs in some cases of phthisis. This variety was often exceedingly distressing to the patient, not only disturbing his sleep at night, and thus wearing him out, but also occasionally bringing on hæmoptysis, and was often associated with sickness. The cough of consumption might be divided into that of phthisis in the first, second, or third stage; or into the cough of active and that of arrested phthisis. Again, cough may be divided according to the position of the lesions, whether these were basic or apical. In the present lecture the subject of cough was divided into different heads according to the time at which it occurred, as follows: 1, evening cough; 2, cough on going to bed; 3, per-noctem cough; 4, cough of early morning; 5, cough after meals; 6, severe cough at any time.

1. Commencing with the evening cough. Patients suffering from this variety when visited in the evening are frequently found sitting in a room, overheated, possibly containing tobacco smoke, engaged in conversation, and may be, complaining that their cough is bad and nothing does it any good. In these patients the following points will be ascertained: that they are weary and tired; the cough is bad; and their temperature is high. In treating these cases the first point to take into consideration is the circumstance in which the patient is living. In phthisis, the night is the most trying time, often the cough is then severe, and the perspirations profuse. In the evening time, therefore, the patient should be prepared for what is usually the worst period of the twenty-four hours. He should, therefore, be rescued from these conditions and sent to bed, which treatment is much more reliable than the giving of drugs. A patient with active phthisis should retire to bed at eight, or at the latest at nine o'clock. As the result of observations made by Dr. Bruce in the Hospital for Consumption, he had found that in cases with high evening temperature, if these be sent to bed early, the temperature frequently falls instead of rises in the later part of the evening. In other words, if the temperature of a patient who is up be taken, say, at eight o'clock, and found to be high, and the following night he be sent to bed at six o'clock, the temperature taken at eight, it will frequently be found to be lower than at the corresponding time on the previous night. The rule Dr. Bruce recommended, was, to send the patients to bed at eight or nine, according to urgency, and as much before these hours if the patient feels tired.

2. Cough on going to bed. In some patients as soon as they go to bed they begin to cough. Probably the chief causes of this variety of cough are due to the movements of undressing, change of temperature, alteration of position, and endeavouring to go to sleep. Here is a definite condition now for treatment. First, send the patient to bed, telling him, never to hurry; all his movements should be slowly performed, and, if necessary, he should be carried to bed. In cold weather there should be a fire in the bed-room, and the bed should be warmed. The patient must be slowly undressed, and clad in a long woollen night-dress. In the case of women some one should do their hair for them, movements of the arms being bad. They should not hurry, but slip into bed, no attempt should be made to lie down at once, but they should first be supported with pillows, or a bed rest, and then slowly lie down.

Thus change of position and all movements are to be regulated. If these points be carefully attended to, the cough will be much less. A companion may sit by the bed and read some not too interesting book, until the patient falls to sleep. If in spite of all these circumstances sleep should refuse to come, then morphine in some form may be administered. This is usually given in a linctus, that is to say a tea-spoonful dose of a thick tenacious substance, which should be slowly sucked down and thus allowed to act on the throat and upper respiratory passages before it is finally absorbed from the stomach. The following is a convenient form of morphia-linctus: Liquoris Morphinæ Acetatis, min. 8; Ætheris Chlorici, min. 3; Succi Limonis, min. 15; Mucilaginem Acaciæ, ad. fl. drm. 1.

3. Cough in the night. In the first place the causes of nocturnal cough are the following:—1, The accumulation of expectoration; 2, exhaustion and irritability of the nerve centres; 3, pyrexia and restlessness. The treatment under these circumstances must be of this kind:—The patient should be allowed to wake up to cough and expectorate. After having coughed and cleared his lungs his strength should be maintained by the administration of nourishment, some warm food, a definite quantity of warm liquid food, such as milk, or broth, or if preferred, cold beef-tea. In addition to warm feeding some stimulant may be given, such as whisky, rum, brandy, or even port wine; of these probably the pure spirit is the best, given with milk. If the patient is having beef-tea the spirit may be given in water afterwards. If, in spite of the feeding, the patient is still sleepless a small dose of morphia-linctus may be repeated.

4. Morning cough. The main points in the treatment of this are—firstly, allow the patient to clear out his lungs; secondly, feed him; thirdly, avoid giving sedatives. In hospitals patients usually have their breakfasts at five o'clock, so also private patients of this description should have an early breakfast. This should consist of warm nutrient wholesome food, such as warm milk, or milk and rum, or warm cocoa with bread and butter, possibly an egg in some form. Often after an early breakfast there will be a short sleep before getting up. A patient with phthisis must learn to go bed at eight or nine, and to rise at eight or nine in the morning. He should not be allowed to lie about in bed or in the house, but should get out into the air if the weather at all permits.

5. Severe cough occurring after meals. This form frequently ends in vomiting. Patients troubled with this sort of cough are usually irritable nervous subjects, with some digestive derangement, together with one or more cavities in the lungs, with fibroid walls. These patients have irritable nervous centres and thus it comes to pass that the respiratory centre being exhausted, stimuli readily overflow from this to the neighbouring gastric centre, hence the vomiting; besides this there may be some special irritability of the pharynx itself. The treatment for this variety of cough consists in rest. The patient should be induced to lie on a couch for an hour or so after meals, keeping perfectly still, until the time for cough and vomiting has passed. In some cases the meals should be taken in a reclining position and it may even be necessary to confine the patient to bed for a time. The second point is a careful regulation of the diet, which should be light, wholesome, and nourishing, being careful to avoid substances liable to produce cough, especially beer. Thirdly, prepare the stomach for the meal before it is taken, either by giving an alkaline or bitter stomachic, ten minutes before meals, or by giving ten grains of a preparation of bismuth in powder. Another good plan is to recommend the patient to lie down for half an hour before meals. He should not come in from a walk and immediately take food, but should first lie down and keep quiet. Again, another plan is to treat what may be the cause of this cough. If there be a large vomica in one of the lungs a blister or counter irritation over the cavity may be serviceable. In some cases great success follows counter irritation over the epigastrum.

6. Severe cough at any time. For this the patient should swallow teaspoonfuls of crushed or powdered ice, or sip cold water. Local applications applied to the throat itself are sometimes advantageous. For this menthol, either in the form of a spray or injected into the back of the throat, or on a sponge as an inhalation, is extremely useful. So, again, menthol lozenges, or warm astringent sprays such as alum. In very bad cases morphine sprays may be used. In severe laryngeal cases insufflation of a twelfth of a grain of acetate of morphia rubbed up with two or three grains of starch is good. In some cases morphine-linctus must be resorted to. In reviewing the lecture Dr. Bruce remarked that one principle had been followed all through, namely, a careful study of the times at which the cough occurs, and not the ordering of a routine cough mixture, which the patient takes as and when he likes.

